# Manipulating the Crystalline Morphology in the Nonfullerene Acceptor Mixture to Improve the Carrier Transport and Suppress the Energetic Disorder

**DOI:** 10.1002/smsc.202100092

**Published:** 2021-11-05

**Authors:** Ming Zhang, Lei Zhu, Chaoqun Qiu, Tianyu Hao, Yufeng Jiang, Shifeng Leng, Jiajun Chen, Guanqing Zhou, Zichun Zhou, Yecheng Zou, Xuan Su, Zhiwen Shi, Haiming Zhu, Yongming Zhang, Thomas P Russell, Xiaozhang Zhu, Feng Liu

**Affiliations:** ^1^ School of Chemistry and Chemical Engineering, Frontiers Science Center for Transformative Molecules, In-situ Center for Physical Science, and Center of Hydrogen Science Shanghai Jiao Tong University Shanghai 200240 P. R. China; ^2^ Department of Polymer Science and Engineering University of Massachusetts Amherst MA 01003 USA; ^3^ Beijing National Laboratory for Molecular Sciences, CAS Key Laboratory of Organic Solids, Institute of Chemistry Chinese Academy of Sciences Beijing 100190 P. R. China; ^4^ Department of Chemistry Zhejiang University Zhejiang 310027 P. R. China; ^5^ State Key Laboratory of Fluorinated Functional Membrane Materials Dongyue Future Hydrogen Energy Materials Company Zibo Shandong 256401 P. R. China

**Keywords:** grazing incidence wide-angle X-ray diffraction, heterojunction thin-film morphologies, nonfullerene acceptors, organic photovoltaics

## Abstract

Mixtures of nonfullerene acceptors (NFAs) are prepared to fine‐tune bulk heterojunction (BHJ) thin‐film morphologies. The acceptor phase resulting from these mixtures has unique physical properties with excellent optoelectronic processes that dictate the output of organic photovoltaic (OPV) devices. Remarkable short‐circuit current densities (*J*_SC_) and fill factors (FFs) are achieved due to the formation of better crystalline fibrils that suppress geminate recombination, leading to improved charge transport with enhanced crystallinity and aligned cascading energy levels confirm efficient exciton diffusion and dissociation, yielding more effective exciton recycling. The decreased Urbach energy and suppressed energetic disorder account for the improvement in the open‐circuit voltage (*V*_OC_). A maximum power conversion efficiency of 17.86% is obtained, underscoring the importance of using specific material interactions to produce a suitable morphology and manage energy loss, resulting in ideal organic solar cell (OSC) devices.

## Graphical abstract

Ternary organic solar cells are fabricated, achieving a significant power conversion efficiency (PCE) improvement, by virtue of an optimized crystalline feature. The optimal morphology ensures efficient exciton diffusion and dissociation, increases the charge transport with reduced recombination, and suppresses energetic disorder, yielding a high efficiency of 17.86%.

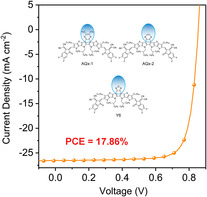

## 1 Introduction

Using mixtures of the nonfullerene acceptors (NFAs) having similar chemical structure to produce multicomponent bulk heterojunction (BHJ) organic solar cells (OSCs) is a promising approach to seek higher power conversion efficiencies (PCEs) by nanostructure optimization and photophysical process refinement.^[^ ^1^, ^2^, ^3^, ^4^, ^5^, ^6^, ^7^ ^]^ The generation of new acceptor phases through intermolecular interactions leads to unique photoelectric properties, avoiding tradeoff between the open‐circuit voltage (*V*_OC_) and short‐circuit current (*J*_SC_), improving device efficiencies.^[^ ^8^, ^9^ ^]^ With state‐of‐the‐art PM6 (poly[(2,6‐(4,8‐bis(5‐(2‐ethylhexyl‐3‐fluoro)thiophen‐2‐yl)‐benzo[1,2‐b:4,5‐b′]dithiophene))‐alt‐(5,5‐(1′,3′‐di‐2‐thienyl‐5′,7′‐bis(2‐ethylhexyl)benzo[1′,2′‐c:4′,5′‐c′]dithiophene‐4,8‐dione)]):Y6 (2,2′‐((2Z,2′Z)‐((12,13‐bis(2‐ethylhexyl)‐3,9‐diundecyl‐12,13‐dihydro‐[1,2,5]thiadiazolo[3,4‐e]thieno[2″,3″:4′,5′]thieno[2′,3′:4,5]pyrrolo[3,2‐g]thieno[2′,3′:4,5]thieno[3,2‐b]indole‐2,10‐diyl)bis(methanylylidene))bis(5,6‐difluoro‐3‐oxo‐2,3‐dihydro‐1H‐indene‐2,1‐diylidene))dimalononitrile) high‐efficiency blends, the unique crystalline nature of Y6 and the fibrillar morphology of PM6 with the size scales compatible with critical optoelectronic processes provides a novel platform for efficient photovoltaic operation.^[^ ^10^, ^11^, ^12^, ^13^, ^14^ ^]^ To explore performance limits, the appropriate combination of materials in generating the mixed acceptor phase is key to improving light absorption, enhancing exciton splitting, and facilitating carrier transport. Carrier generation processes must shunt energy loss channels induced by low energy charge transfer (CT) states to improve *V*_OC_,^[^ ^15^, ^16^, ^17^ ^]^ which is essential for optimizing OSC devices.

In this study, mixtures of two NFAs, Y6:AQ*x*−1 (2,2′‐((2Z,2′Z)‐((13,14‐bis(2‐ethylhexyl)‐6,7‐dimethyl‐3,10‐diundecyl‐13,14‐dihydrothieno[2″,3″:4′,5′]thieno[2′,3′:4,5]pyrrolo[3,2‐f]thieno[2″,3″:4′,5′]thieno[2′,3′:4,5]pyrrolo[2,3‐h]quinoxaline‐2,11‐diyl)bis(methaneylylidene))bis(5,6‐difluoro‐3‐oxo‐2,3‐dihydro‐1H‐indene‐2,1‐diylidene))dimalononitrile) and Y6:AQ*x*−2 (2,2′‐((2Z,2′Z)‐((13,14‐bis(2‐ethylhexyl)‐3,10‐diundecyl‐13,14‐dihydrothieno[2″,3″:4′,5′]thieno[2′,3′:4,5]pyrrolo[3,2‐f]thieno[2″,3″:4′,5′] thieno[2′,3′:4,5]pyrrolo[2,3‐h]quinoxaline‐2,11‐diyl)bis(methaneylylidene))bis(5,6‐difluoro‐3‐oxo‐2,3‐dihydro‐1H‐indene‐2,1‐diylidene))dimalononitrile), described in detail later, were used. Improved acceptor phase crystallization was achieved, and the crystallization of the donor was also enhanced, yielding more balanced charge transport. More efficient exciton diffusion and dissociation were found, promoting optoelectronic processes to improve *J*_SC_'s by more efficient exciton cycling. The decreased Urbach energy and suppressed energetic disorder contributed the elevation of *V*_OC_. With the PM6:Y6 blend platform, simultaneous improvements of the *V*_OC_, *J*_SC_, and FF were realized, resulting in a PCE of 17.86%.

## 2 Results and Discussion

**Figure 1:**
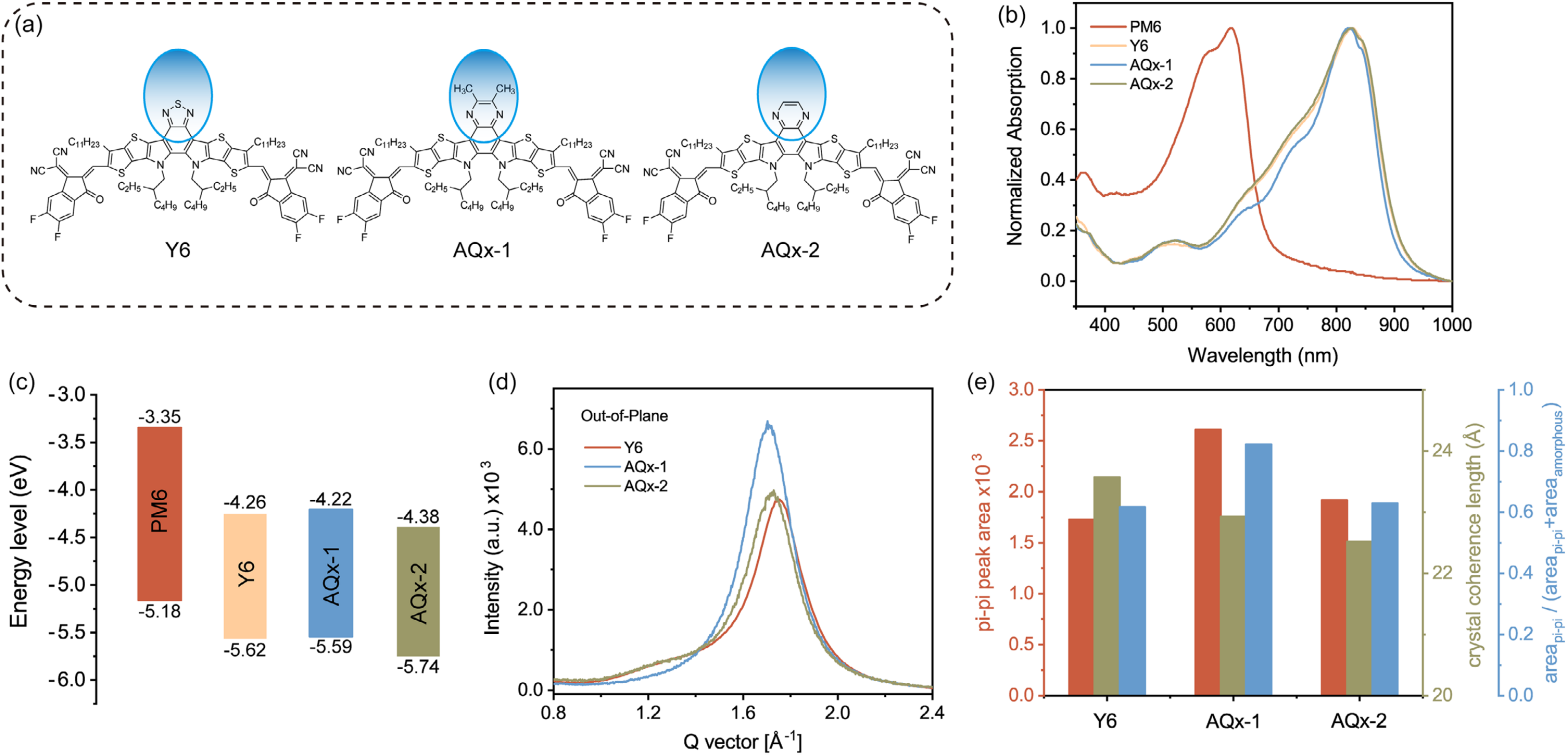
a) Chemical structures, b) normalized thin‐film absorption, and c) energy level diagram of the materials used in the experiment. d) OOP GIWAXS line cuts of the neat films, and e) the corresponding fitting results, including peak area (red) reflecting the amount of the crystallites, CCL (dark yellow) representing the crystalline quality, and the diffraction area division reflecting the relative crystallinity.

The molecular structures of the materials used in the ternary blend OSCs are shown in **Figure** **1**a. In comparison to Y6, the modulation of the substitution group along the conjugated framework affords AQ*x*−1 and AQ*x*−2 different electronic structures, crystallization behavior, and miscibility in the blended thin films.^[^ ^18^ ^]^ The normalized UV–vis absorption spectra of the neat thin films are shown in Figure 1b. Y6 shows a strong, broad absorption from 700 to 900 nm with a peak at 828 nm. AQ*x*−2 shows a similar absorption, while the absorption tail of AQ*x*−1 is slightly blue shifted. The main absorption of PM6 occurs from 400 to 700 nm, complementing that of the acceptors. The energy levels of the materials were determined by ultraviolet photoelectron spectroscopy (UPS).^[^ ^19^, ^20^ ^]^ By fitting the intensity onset of the frontier electronic structure region and the secondary electron cutoff (Figure S1a, Supporting information), the highest occupied molecular orbital (HOMO) level referenced to the vacuum energy level (*E*_vac_) is determined. The lowest unoccupied molecular orbital (LUMO) level could be obtained by fitting the absorption edge of the neat films to extract the optical bandgap. The corresponding energy levels are diagrammed in Figure 1c and Figure S1b, Supporting information. The HOMO and LUMO levels determined for AQ*x*−1 and AQ*x*−2 thin films were −5.59/−4.22 and −5.74/−4.38 eV, respectively. The crystalline properties were investigated by grazing incidence wide‐angle X‐ray scattering (GIWAXS) measurement. The 2D patterns of the neat films prepared from chloroform (CF) solutions are shown in Figure S2, Supporting information. All the acceptors adopt a face‐on orientation with the π−π stacking peak in the out‐of‐plane (OOP) direction, and lamellar peak in the in‐plane (IP) direction. AQ*x*−1 shows the best order with enhanced (110) and (11‐1) diffraction signals. The OOP line cuts in Figure 1d show the π−π stacking peak and the amorphous halo at ≈1.3 Å^−1^. AQ*x*−2 and Y6 share similar diffraction intensities for the π−π stacking peak and the amorphous halo, indicating a similar degree of crystallinity. For the AQ*x*−1 thin film, the π−π stacking peak is more pronounced, while the amorphous halo is significantly suppressed (almost vanishing), indicating the improved crystallinity of AQ*x*−1 thin film. The diffraction signals in 1.0–2.4 Å^−1^ region were fit with multiple Lorentzians to quantitively extract the peak area and the full width at half maximum (FWHM). The results are shown in Figure 1e. The crystal coherence length (CCL), calculated from the FWHM using the Scherrer equation,^[^ ^21^, ^22^ ^]^ is commonly used to define the persistence of the crystalline lattice in the direction defined by the reflection. The peak area, relative to the total scattering, directly reflects the degree of crystallinity. Using only the scattering vector range for the π−π stacking reflection, a relative degree of crystallinity was determined. Y6 shows the largest persistence length, but the difference is not very significant (1.0–2.0 Å), as the dark yellow bar indicates in Figure 1e. AQ*x*−1 shows the highest crystallinity, while AQ*x*−2 shows a similar degree of crystallinity as Y6, indicative of the different crystalline features arising from the substitution groups.

**Figure 2:**
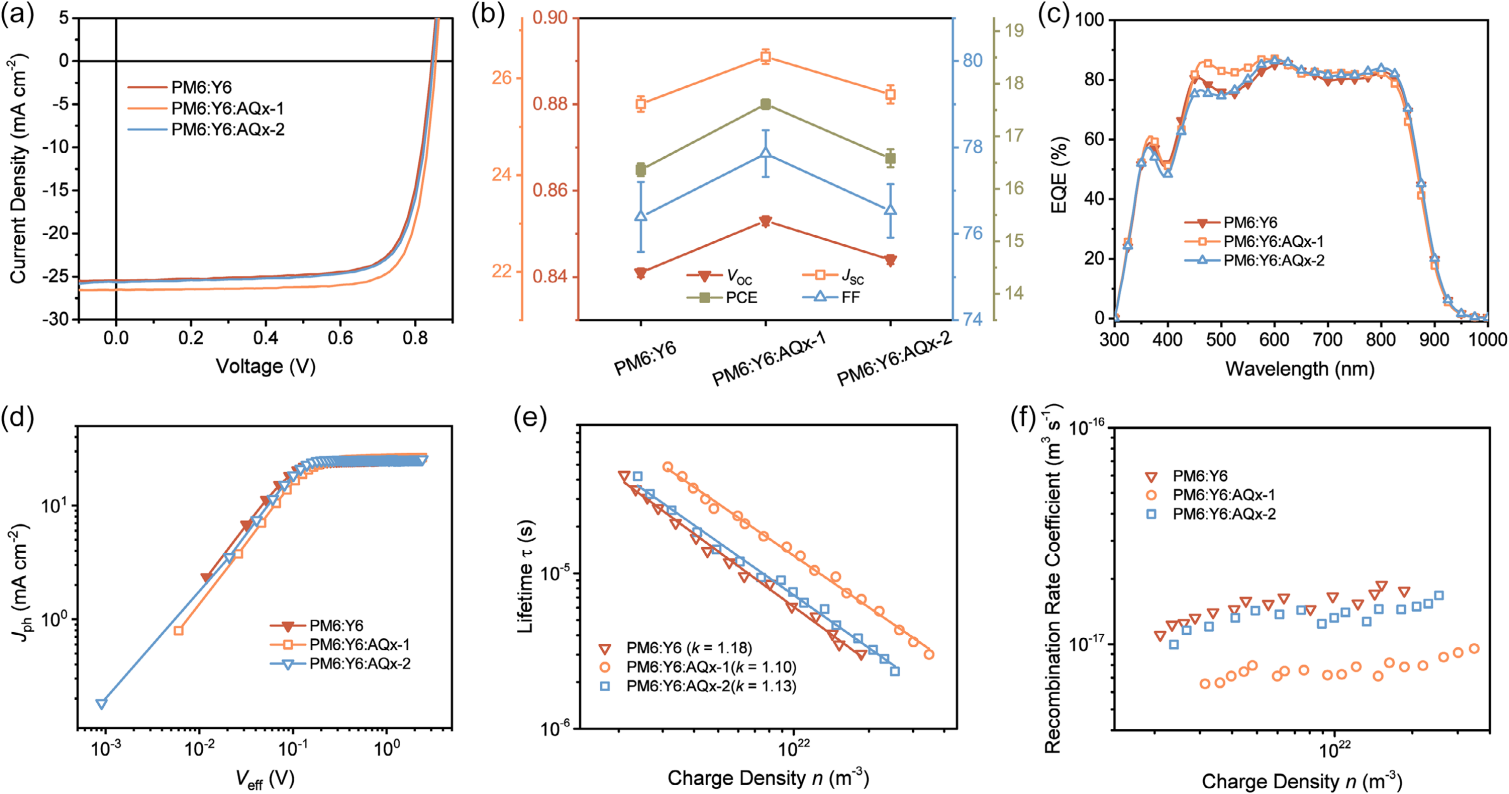
a) Current density–voltage characteristics, b) *V*_OC_, *J*_SC_, FF, PCE, and c) external quantum efficiency of the optimal devices. d) Photocurrent density (*J*_ph_) versus effective bias (*V*_eff_) characteristics and e) Charge lifetime in the devices as a function of charge density. The solid lines represent a best fit to power law dependence with a slope of *k*. f) Measured nongeminate recombination rate coefficient for devices.

The photovoltaic characteristics in ternary OSCs based on AQ*x*−1 and AQ*x*−2 NFAs were measured. The devices were fabricated with the conventional architecture of indium‐tin‐oxide (ITO)/poly(3,4‐ethylenedioxythiophene):poly(styrenesulfonate) (PEDOT:PSS)/active layer/PFNDI‐Br/Ag. For the binary OSCs based on PM6:Y6 and PM6:AQ*x*−1, the active layers were spin‐cast from CF with a donor/acceptor weight ratio of 1:1.2 containing 0.5% (vol%) 1‐chloronaphthalene (1‐CN) as an additive. The ternary OSCs based on PM6:Y6:AQ*x*−1 and PM6:Y6:AQ*x*−2 blends were fabricated using similar procedures with the weight ratio of PM6:acceptors fixed at 1:1.2, following the optimized conditions for the binary OSCs, to avoid light‐absorption‐induced performance change. The current density–voltage (*J*–*V*) curves of the optimized devices are shown in **Figure** **2**a and the corresponding device parameters are summarized in Figure 2b and **Table** **1**. The PM6:Y6 device produced an average PCE of 16.36% with a *V*_OC_ of 0.841 V, a *J*_SC_ of 25.47 mA cm^−2^,and an FF of 76.39%. Devices based on PM6:AQ*x*−1 yielded a PCE of 14.33%, with a *V*_OC_ of 0.875 V, a *J*_SC_ of 22.68 mA cm^−2^, and an FF of 72.22%. For PM6:Y6:AQ*x*−1 devices, an impressive average PCE of 17.61% (maximum PCE of 17.86%) was produced with an average *V*_OC_ of 0.853 V, an average *J*_SC_ of 26.45 mA cm^−2^, and an average FF of 77.86%, while a slightly enhanced average PCE of 16.58% was obtained for the PM6:Y6:AQ*x*−2 devices. The detailed device parameters for the different mixing ratios are shown in Table S1 and S2, Supporting Information. The best PM6:Y6:AQ*x*−1 device gave a certified PCE of 17.20% (National Institute of Metrology using a 3.152 mm^2^ photon mask) as shown in Figure S3, Supporting Information. External quantum efficiency (EQE‐PV) spectra of the optimized binary and ternary OSCs are shown Figure 2c, with relative EQE shown in Figure S4, Supporting Information. The PM6:Y6 device had a broad photoresponse from 300 to 950 nm. With AQ*x*−1 a high and flat profile at an EQE‐PV over 80% from 460 to 800 nm is seen for the ternary devices, reflecting excellent photomanagement over multiple components. Thus, the addition of AQ*x*−1 increases the EQE from 450 to 600 nm, complementing the absorption dip in PM6:Y6, leading to more efficient photon extraction. The decrease in the EQE from 800 to 950 nm arises from the blueshift of the absorption tail. Similar behavior is seen for the PM6:Y6:AQ*x*−2 system, but not pronounced. The photocurrent (*J*_ph_), as a function of the effective voltage (*V*_eff_), was measured, with *V*_eff_ = *V*_0_ − *V*_appl_, (*V*_appl_ is the applied voltage, *J*_ph_ = *J*_L_−*J*_D_ where *J*_L_ and *J*_D_ are current density under illumination and dark conditions, respectively, and *V*_0_ is the voltage when *J*_ph_ = 0 mA cm^−2^), as shown in Figure 2d. The charge dissociation probabilities (defined as *P* (*E*, *T*), where *E* is the electric field *E* and *T* is the temperature) were calculated based on the normalized *J*_ph_ in the short‐circuit current condition with respect to the saturated photocurrent at high reverse bias, where *P* (*E*, *T*) technically approaches 100%. *P* (*E*, *T*) values of 98.1%, 99.6%, and 98.6% for the PM6:Y6, PM6:Y6:AQ*x*−1, and PM6:Y6:AQ*x*−2 device, respectively, were obtained, indicating that the addition of AQ*x*−1 into the binary blend slightly promotes charge generation and exciton dissociation. The dependence of the *J*_SC_ on the light intensity is shown in Figure S5a, Supporting information. The correlation between *J*_SC_ and light intensity (*P*_in_) can be expressed as $J_{\text{SC}}\approx P_{\text{in}}^{\alpha }$, where *α* is close to 1 suggesting minimal bimolecular recombination. The *α* values were 0.974, 0.995, 0.987 for PM6:Y6, PM6:Y6:AQ*x*−1 and PM6:Y6:AQ*x*−2 devices, respectively. The relatively large *α* approaching unity suggests a suppressed bimolecular recombination in optimized ternary active layers, which agrees well with the relatively high FF of the related ternary PSCs. The dependence of *V*_OC_ on light intensity (*P*_light_) was examined to further understand the recombination processes. Generally, with a slope close to *kT*/q (where *k* is the Boltzmann constant, *T* is the temperature in Kelvin, *q* is the elementary charge), trap‐assisted recombination should be negligible. As shown in Figure S5b, Supporting Information, the slopes are found to be 1.092 *kT*/q, 1.056 *kT*/q, and 1.085 *kT*/q for PM6:Y6, PM6:Y6:AQ*x*−1, and PM6:Y6:AQ*x*−2 devices, respectively, indicating that, in the ternary devices, trap‐assisted recombination is suppressed., The hole (*μ*_h_) and electron (*μ*_e_) mobilities in the binary and ternary blends were determined from space charge limited current (SCLC) measurements (Figure S6,S7 and Table S3, Supporting Information). Both the electron and hole mobilities improve, indicating successful management of free carriers over the multiple components in the ternary blends, which agrees well with the increases in *J*_SC_ and FF.^[^ ^23^ ^]^ To elucidate the influence of the AQ*x*−1 and AQ*x*−2 NFAs on the charge transport properties in detail, transient photovoltage (TPV) and transient photocurrent (TPC) measurements were applied. Lifetimes under different *V*_OC_ conditions (tuned by changing the light intensity) could be obtained through exponential decay fitting, as shown in Figure S8a, Supporting Information. The lifetime of the ternary devices shows an obvious enhancement in the whole *V*_OC_ regime, especially PM6:Y6:AQ*x*−1 device, indicating optimized device condition. The charge density as a function of *V*_OC_ for devices is calculated by differential capacitance method, with results shown in Figure S8b, Supporting Information. The improved charge density in the ternary devices echo promoted quantum yield with efficient transfer and transport. Derived charge lifetime in the devices as a function of charge density is shown in Figure 2e, following a power law dependence, indicating the nongeminate recombination is the dominating loss channel for carrier density under open‐circuit condition. The recombination order *R* is determined by the exponential factor *k* (*R* = *k* + 1, and *k* is the slope of the exponential fitting). In the case of strictly bimolecular recombination, *R* is expected to be 2. A higher value of *R* (*R* > 2) reveals the presence of trap states introduced by defects and inherent disordering in molecular packing that could contribute to carrier recombination.^[^ ^24^, ^25^ ^]^ And in our case, the decreased *R* values in the ternary devices indicate suppressed defects and trap states, agreeing well with the light‐dependent measurements (Figure S5, Supporting Information). According to the above, nongeminate recombination rate coefficient can be determined, which is defined by $\frac{1}{\tau \left(n\right)n}$, as shown in Figure 2f, where suppressed recombination is clearly seen in the ternary devices. The higher mobilities and reduced recombination during the transport contribute to improved devices photoelectric properties.

**Table 1:** Summary of photovoltaics of binary and ternary solar cells under illumination of AM 1.5G, 100 mW cm^−2^

Blend	*V*_OC_ [V]	*J*_SC_ [mA cm^−2^]	FF [%]	PCE [%]
PM6:Y6a)	0.841 ± 0.002	25.47 ± 0.16	76.39 ± 0.81	16.36 ± 0.12
PM6:Y6:AQ*x*−1a)	0.853 ± 0.003	26.45 ± 0.15	77.86 ± 0.54	17.61 ± 0.09 (17.86)b)
PM6:Y6:AQ*x*−2a)	0.844 ± 0.003	25.67 ± 0.19	76.53 ± 0.62	16.58 ± 0.17
PM6:AQ*x*−1a)	0.875 ± 0.001	22.68 ± 0.28	72.22 ± 0.93	14.33 ± 0.28
PM6:AQ*x*−2a)	0.858 ± 0.004	25.27 ± 0.27	76.01 ± 1.01	16.33 ± 0.24

a) Average values are obtained from 40 devices;

b) Maximum values are obtained.

**Figure 3:**
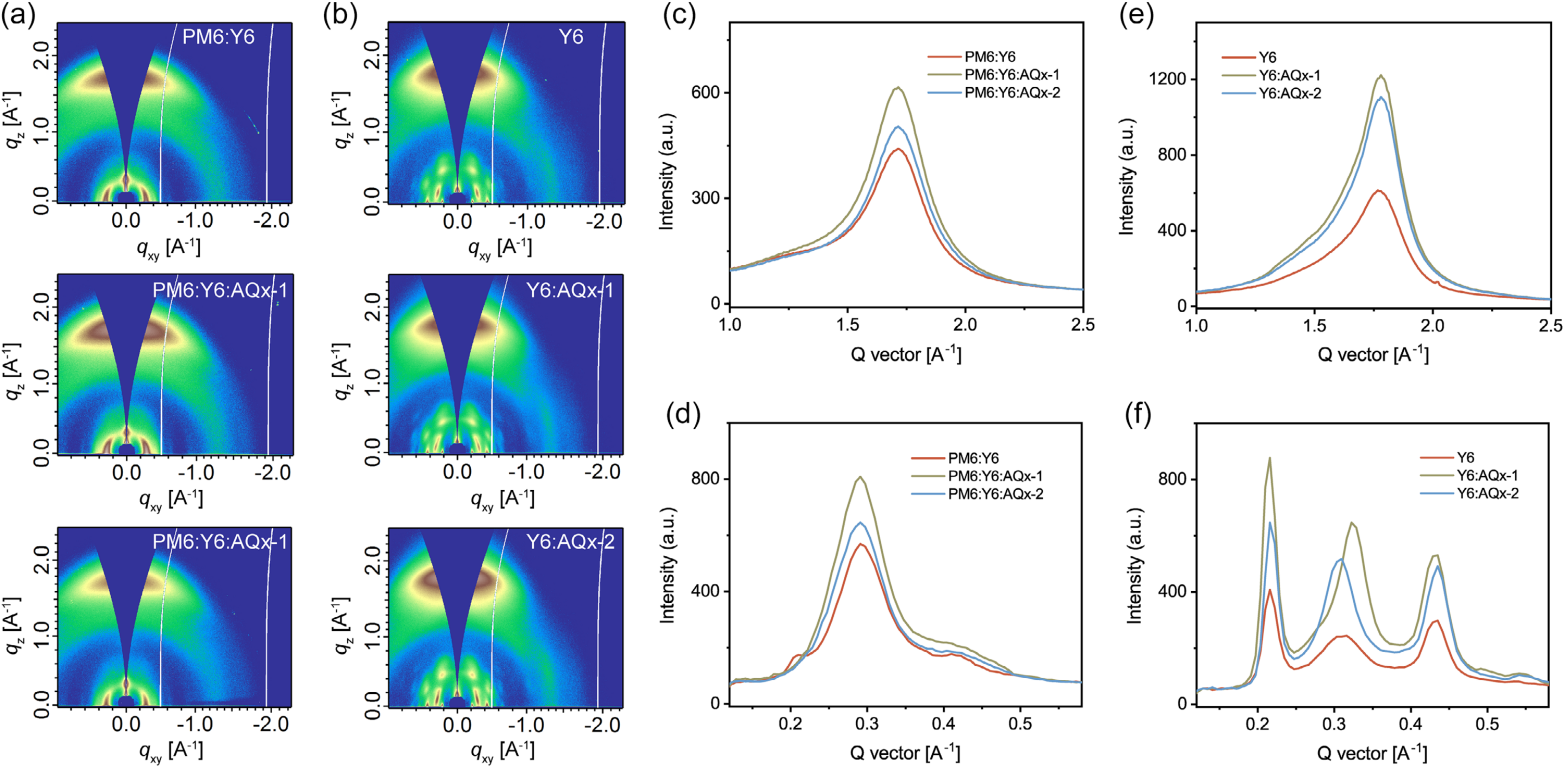
2D GIWAXS patterns for a) BHJ and b) acceptor‐only thin films. c) The large‐q region of the OOP line cuts and d) the small‐q region of the IP line cuts for the BHJ thin films. e) The large‐q region of the OOP line cuts and f) the small‐q region of the IP line cuts for the acceptor‐only thin films.

The structure of the BHJ and the acceptor‐only (Y6:AQ*x*−1 and Y6:AQ*x*−2) thin films were determined using GIWAXS, with 2D patterns shown in **Figure** **3**a,b. In the BHJ blends, PM6 dominates, keeping a preferential face‐on orientation, as evidenced by the pronounced (010) diffraction peak in the OOP direction. The crystallization of the acceptors is significantly suppressed, where the (020), (11‐1), and (021) diffraction essentially vanish. In the acceptor‐only thin films, the crystallization of the acceptors is maintained, as evidenced by the bright scattering spots in the low‐q region, and the π−π stacking peak with typical shape in the OOP direction. After the addition of AQ*x*−1 and AQ*x*−2, the q‐position is maintained, but there are intensity differences. The (020) and (11‐1) reflections are located in‐plane (azimuthal angle of 0^°^), while the (110) reflection is seen at an azimuth angle of 25^°^. Consequently, when dealing with the BHJ thin films, a 0^°^ (IP) line cut with a 10^°^ width was used to obtain the diffraction profiles of PM6, avoiding overlap with the Y6 signal, even though it is weak. Thus, the crystallization of PM6 could be directly determined from the GIWAXS results of the BHJ thin films, and the crystallization of the acceptors could be estimated from the results of the acceptor‐only films. Figure 3c is the high‐q region of the OOP line cuts. After the addition of AQ*x*−1 and AQ*x*−2, the intensity of the peak increases. Figure 3d is the low‐q region IP line cut of the BHJ thin films, where, as discussed above, the lamellae peak arises only from PM6, confirming enhanced crystallization of the PM6 in the ternary blends, which accounts for the increase in the EQE from 450–600 nm region. The results of the acceptor‐only films are shown in Figure 3e,f. The addition of a small amount of AQ*x*−1 and AQ*x*−2 almost doubles the intensity of the π−π stacking peak, and the (110) peak in the IP direction emerges with an obvious change in the peak position, indicating the emergence of a new structure where the packing motif is maintained but the packing distance changes. The similarity of NFA molecules leads to an intimate mixing and coassembly with better order. The morphologies of the blends were also characterized by transmission electron microscopy (TEM) and atomic force microscopy (AFM), as shown in Figure S9 and S10, Supporting information. All the BHJ thin films showed evidence of phase separation on the tens of nanometers length scale with low surface mean‐square surface roughnesses (*R*_q_) (minimum value of 0.699 nm).

**Figure 4:**
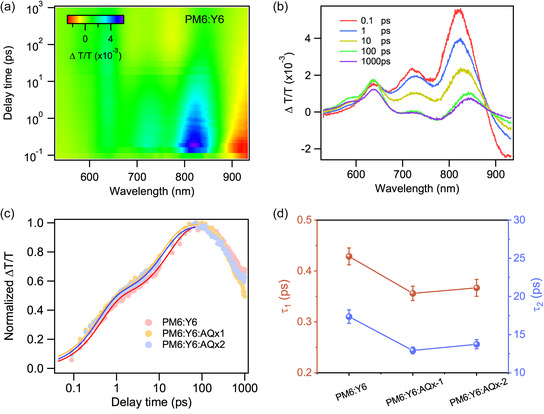
a) Color plot of fs transient absorption spectra of blended film at indicated delay times under 750 nm excitation with a fluence below 10 μJ cm^−2^ and b) representative fs TA spectra of blended films at indicated delay times. c) TA kinetics of hole transfer process for the blended films (solid circles) and corresponding bi‐exponential fitting (solid line); d) CT time achieved through multiexponential fitting for different blended films.

The carrier transfer dynamics of blends were investigated by femtosecond transient absorption (TA) spectroscopy to probe the photo‐induced hole transfer dynamics directly in the blended films. The results are shown in **Figure** **4**, with the corresponding hole transfer times summarized in Table S4, Supporting Information. An excitation wavelength of 750 nm was used to selectively excite the acceptors. The 2D color map TA spectra of the blend films after 750 nm excitation are shown in Figure 4a and Figure S11, Supporting Information, with a few representative TA spectra at the indicated delay times shown in Figure 4b. The bleach peaks around 850 nm appeared in both neat Y6 film and Y6‐based binary and ternary blend films, corresponding to the ground‐state bleach (GSB) and stimulated emission of absorption transition in Y6 due to photoexcitation. With the decay of the Y6 bleach peak at 700–850 nm, a few clear bleach peaks at 550–630 nm appeared in the TA spectrum of the blend film, matching well with the absorption features of neat PM6 films. Using a bi‐exponential fitting, the ground‐state bleach of PM6 at 595 nm in the binary and ternary blends (PM6:Y6, PM6:Y6:AQ*x*−1, and PM6:Y6:AQ*x*−2) increased with half‐times *τ*_1_ of ≈0.43, ≈0.36, ≈0.37 ps, and *τ*_2_ of 17.4, 12.9, 13.7 ps, respectively. The former fast component *τ*_1_ can be assigned to the ultrafast exciton dissociation of the acceptors at the donor–acceptor interface, and the slow component τ_2_ to the diffusion of excitons in the acceptors toward the interface before dissociation, one order of magnitude larger than the exciton dissociation.^[^ ^26^, ^27^, ^28^ ^]^ It is interesting to see the hole transfer process is independent of the HOMO energy offset, considering the countering contributions of AQ*x*−1 and AQ*x*−2 to this driving force. A cascading energy ladder may account for this behavior.^[^ ^14^, ^29^ ^]^ From a morphological viewpoint, the presence of the secondary acceptor induces improved long‐range order structurally to form a sharp domain interface, fundamentally promoting the optoelectronic process to yield an improved *J*_SC_ by more efficient exciton recycling.

**Figure 5:**
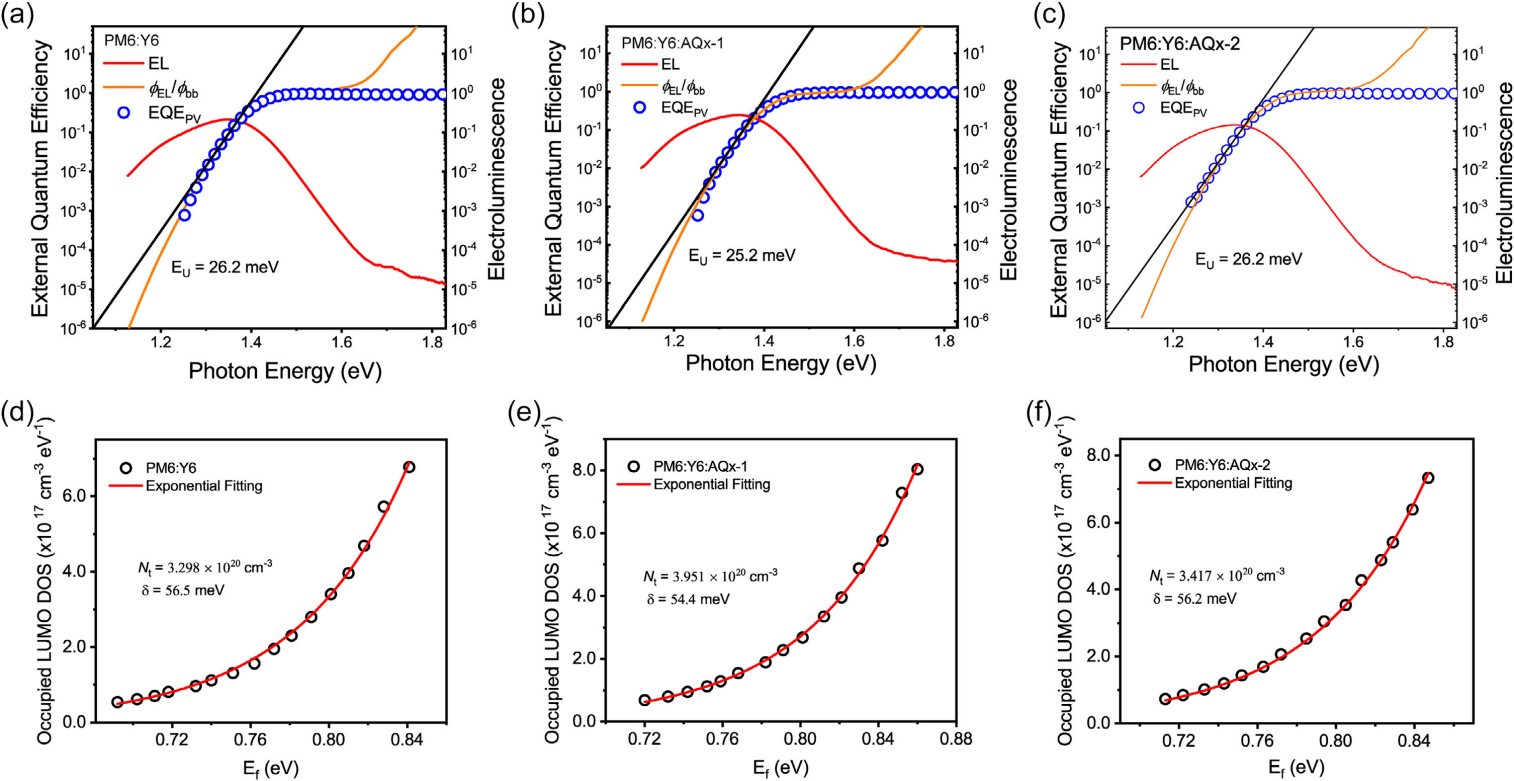
a–c) The semilogarithmic plots of the normalized EL (red solid line), normalized EQE (blue open circles) and the division of electroluminescence (*Φ*_EL_) and black‐body emission (*Φ*_bb_) (orange solid line) of the PM6:Y6, PM6:Y6:AQ*x*−1 and PM6:Y6:AQ*x*−2 devices. The black solid line is the Urbach energy fitting. d–f) The derived LUMO DoS from the capacitance spectra of PM6:Y6, PM6:Y6:AQ*x*−1, and PM6:Y6:AQ*x*−2 devices, exhibiting an exponential shape, where *N*_t_ is the total density per unit volume, and *δ* the energetic disorder parameter.

We further explored the impact of the excess acceptors on the *V*_OC_ loss in the fabricated devices, where the *V*_OC_ increase is mainly ascribed to the filling state management, and suppressed energetic disorder. S‐EQE, electroluminescence (EL), and impedance spectroscopy (IS) were performed to investigate *V*_OC_ details.^[^ ^30^, ^31^, ^32^ ^]^ As shown in **Figure** **5**a,b, the Urbach energy (*E*_U_) of the binary and ternary devices could be quantificationally obtained by fitting the low photon energy region of s‐EQE curves to study the radiative recombination loss below the bandgap. The PM6:Y6:AQ*x*−1 film exhibits rather lower energy disorder with an *E*_U_ of 25.2 meV (Figure 5b and Figure S12, Supporting Information), which is smaller than that of PM6:Y6 binary device (26.2 meV). Such a result agrees well with the absorption of the thin films, where a sharper absorption onset is seen in PM6:Y6:AQ*x*−1 blend, indicative of smaller Urbach energy (Figure S13, Supporting Information).^[^ ^33^ ^]^ IS was employed to access the density of state (DOS) information. The chemical capacitors ($C_{\mu }^{n}$) determined by IS (Figure S14, Supporting Information) reflects the capability of the photovoltaic device to accept or release additional charge carriers as the result of the shifting in the quasi‐Fermi level. The DoS (*g*_n_) is obtained by $C_{\mu }^{n}[F\cdot {\text{cm}}^{-3}]=q^{2}\cdot g_{n}(E_{\text{Fn}})[{\text{cm}}^{-3}\cdot {\text{eV}}^{-1}]$, where $C_{\mu }^{n}$ equals to C/(L·S) (*L* and *S* are the thickness and area of the device) and *q* is the elemental charge. A typical exponential shape is seen (Figure 5c–e), and the curves are fitted by $g_{n}(E_{\text{Fn}})[{\text{cm}}^{-3}\cdot {\text{eV}}^{-1}]=\frac{N_{t}\left[{\text{cm}}^{-3}\right]}{\delta \left[\text{eV}\right]}\text{exp}(-\frac{E_{g}-E_{\text{Fn}}\left[\text{eV}\right]}{\delta \left[\text{eV}\right]})$, where $N_{t}$ is the total density per unit volume, $E_{g}$ is the bandgap, and *δ* is the broadening of the DOS that describes energetic disorder. Here, we used $E_{g}^{\text{PV}}$(EQE spectra edge, Figure S15, Supporting Information) as the $E_{g}$, and the fitting results are summarized in Table S5, Supporting Information. As shown in Figure 5c–e, the $N_{t}$ values correlate well with the charge density derived from TPC measurements, thus, a significant *J*_SC_ improvement is seen in PM6:Y6:AQ*x*−1 device. PM6:Y6 device shows a large *δ* of 56.5 meV, and the addition of AQ*x*−1 and AQ*x*−2 could suppress the energetic disorder, especially in PM6:Y6:AQ*x*−1 device with a rather smaller *δ* of 54.4 meV, thus, a high *V*_OC_ is obtained.

## 3 Conclusion

In conclusion, ternary organic solar cells based on two nonfullerene small molecule acceptors, AQ*x*−1 and AQ*x*−2, were designed. Remarkable *J*_SC_ and FF improvement are achieved, with the formation of more crystalline acceptor fibrils, with suppressed bimolecular recombination and trap‐assisted recombination, improving the transport of the free carriers. A much more efficient exciton diffusion and interfacial separation process is achieved, due to structurally enhanced crystallinity and aligned cascading energy levels, yielding more effective exciton recycling. The suppressed energetic disorder and decreased Urbach energy account for the improvement of the *V*_OC_, thus, a maximum PCE of 17.86 % was obtained. The structural similarity of the NFAs AQ*x*−1 and AQ*x*−2 enables the generation of a well‐suited morphological framework, capitalizing on the electronic properties of the materials to manage energy loss, further promoting the photoelectric output.

## 4 Experimental Section

See experimental details in the Supporting Information.

## 6 Conflict of Interest

The authors declare no conflict of interest.

## 7 Data Availability Statement

The data that support the findings of this study are available from the corresponding author upon reasonable request.

## Supporting information

Supplementary Material
