# Supplementary material for: Manipulating the Crystalline Morphology in the Nonfullerene Acceptor Mixture to Improve the Carrier Transport and Suppress the Energetic Disorder
Source: Small Sci. 2021 Nov 5;2(2):2100092. doi: 10.1002/smsc.202100092 (PMC11936009; doi:10.1002/smsc.202100092)
Supplement: Supplementary file 1 — Supplementary Material [file SMSC-2-2100092-s001.pdf]

Supporting Information

Manipulating the crystalline morphology in the non-fullerene acceptor mixture to improve the carrier transport and suppress the energetic disorder

Ming Zhang, Lei Zhu, Chaoqun Qiu, Tianyu Hao, Yufeng Jiang, Shifeng Leng, Jiajun Chen, Guanqing Zhou, Zichun Zhou, Xiaonan Xue, Yecheng Zou, Xuan Su, Zhiwen Shi, Haiming Zhu, Yongming Zhang, Thomas P Russell*, Xiaozhang Zhu*, Feng Liu**

Ming Zhang,[+] Lei Zhu,[+] , Chaoqun Qiu, Tianyu Hao, Shifeng Leng, Jiajun Chen, Guanqing Zhou, Zichun Zhou, Xiaonan Xue, Prof. Zhiwen Shi, Prof. Yongming Zhang, Prof. Feng Liu
School of Chemistry and Chemical Engineering, Frontiers Science Center for Transformative Molecules, In-situ Center for Physical Science, and Center of Hydrogen Science Shanghai Jiao Tong University, Shanghai 200240, P. R. China.
E-mail: fengliu82@sjtu.edu.cn

Yufeng Jiang, Prof. Thomas Russell
Department of Polymer Science and Engineering, University of Massachusetts, Amherst, MA 01003, USA Department of Polymer Science and Engineering, University of Massachusetts, Amherst, MA 01003, USA.
E-mail: russell@mail.pse.umass.edu

Prof. Xiaozhang Zhu
Beijing National Laboratory for Molecular Sciences, CAS Key Laboratory of Organic Solids, Institute of Chemistry, Chinese Academy of Sciences, Beijing 100190, P. R. China.
E-mail: xzzhu@iccas.ac.cn

Prof. Haiming Zhu
Department of Chemistry, Zhejiang University, Zhejiang 310027, P. R. China.
E-mail: hmzhu@zju.edu.cn

Dr. Yecheng Zou, Dr. Xuan Su, Prof. Yongming Zhang, Prof. Feng Liu
State Key Laboratory of Fluorinated Functional Membrane Materials and Dongyue Future Hydrogen Energy Materials Company, Zibo City, Shandong 256401, P. R. China.

[+]These authors contributed equally to this work.

Experimental Section

Materials: All reagents and chemicals were purchased from commercial sources (Adamas-beta) and used without further purification. The silicon nitride membrane is from Clean-SiN Company. PM6, Y6 were purchased from Solarmer Company. The number-average

molecular weight (M_n) and polydispersity index (PDI) of PM6 used in this manuscript were 29.8 kDa and 2.32, respectively. The AQx-1 and AQx-2 is from Xiaozhang Zhu's group.

Methods: UV-vis absorption spectra were recorded on a Shimadzu spectrometer model UV-1800 with films on the quartz plates at room temperature. The morphologies of the nanostructures were characterized by transmission electron microscopy (TEM, JEM-1400, JEOL, Japan). The GIWAXS characterization of the thin films was performed at the Advanced Light Source (Lawrence Berkeley National Laboratory) on beamline 7.3.3. The incidence angle was 0.16°, and the beam energy was 10 keV. Samples were prepared under device conditions on the Si/PEDOT:PSS substrates.

Device Fabrication: Organic solar cell devices with ITO/PEDOT:PSS/Active Layer/PFNDI-Br/Ag structures were fabricated according to the following procedure. Patterned ITO glass substrates were sequential cleaned by ultrasonicating in acetone, detergent, deionized water and isopropyl alcohol for 15 min each and then dried under dry oven. The precleaned substrates were treated in an ultraviolet-ozone chamber for 15 min, then a ~40 nm thick PEDOT:PSS (Clevious P VP AI 4083 H. C. Stark, Germany) thin film was deposited onto the ITO surface by spin-coating and baked at 150 °C for 15 min. The blend solution with different mixing ratio (14.3 mg mL^{-1} in total) in CF (with 0.5% CN solvent additive) was stirred at 25 °C for 120 min in advance and then spin-coated on top of the PEDOT:PSS layer. The prepared films were treated with thermal annealing at 85°C for 5 min. After cooling to room temperature, a ~5 nm thick of PFNDI-Br was spin-coated on the top of active layer. Then, those samples were brought into to an evaporate chamber and a 150 nm thick silver layer was thermally evaporated on the PFNDI-Br layer at a base pressure of 1×10^{-6} mbar. The evaporation thickness was controlled by SQC-310C deposition controller (INFICON,

Germany). Ten devices were fabricated on one substrate and the active area of each device was 0.032 cm^2 defined by a shadow mask.

Device Characterization. The current-voltage (*J-V*) curves were measured with Keithley 236 Source under the illumination of AM 1.5G irradiation (100 mW cm^{-2}) using a 150 W solar simulator (DM-40S3, SAN-EI ELECTRIC, Japan) in glove box at room temperature. The light intensity was determined by a 2 × 2 cm^2 standardized monosilicon cell (Oriel PN 91150V, Newport, USA.) calibrated by the National Renewable Energy Laboratory (NREL). The external quantum efficiency (EQE) measurement was performed using an Enlitech EQE system (Enlitech QE-M110) with a Si diode as reference cell. The *J*−*V* curves were measured along the forward scan direction from -0.1 to 1.0 V or the reverse scan direction from 1.0 to -0.2 V, yielding identical results. The scan speed and dwell times were fixed at 0.015 V s^{-1} and 20 ms, respectively.

SCLC mobility measurements. The electron-only devices were fabricated with ITO/ZnO/Active Layer/ZnO/Ag structures and hole-only devices were fabricated with ITO/PEDOT:PSS/Active Layer/MoO_x/Al structures. The space charge limited current (SCLC) mobility was calculated according to the Mott-Gurney square law $J = 9\varepsilon_r\varepsilon_0\mu V^2/8L^3$, where J is the current density, ε_r is the relative dielectric constant of the transport medium component, ε_0 is the vacuum permittivity, μ is the electron or hole mobility, V is the effective voltage, and L is the thickness of active layer.

Ultraviolet photoelectron spectroscopy measurement. Ultraviolet Photoelectron Spectroscopy analysis was conducted using an AXIS Ultra DLD spectrometer (Kratos Analytical Inc., Mancheser, UK) with a He discharge UV lamp with He I radiation (incident

photo energy, 21.22eV), a hemispherical analyzer operating in the fixed analyzer transmission mode and the standard aperture (analysis area: 0.3 mm * 0.7 mm).

Transient absorption spectroscopy. For femtosecond transient absorption spectroscopy, the fundamental output from Yb:KGW laser (1030 nm, 220 fs Gaussian fit, 100 kHz, Light Conversion Ltd) was separated to two light beam. One was introduced to NOPA (ORPHEUS-N, Light Conversion Ltd) to produce a certain wavelength for pump beam (here we use 550 and 750 nm, 30 fs pulse duration), the other was focused onto a YAG plate to generate white light continuum as probe beam. The pump and probe overlapped on the sample at a small angle less than 10°. The transmitted probe light from sample was collected by a linear CCD array.

Electroluminescence measurement. Electroluminescence spectrum measurement was conducted by direct-current meter (PWS2326, Tectronix) to provide bias voltage for the test device, and the electroluminescence emissions were recorded by the fluorescence spectrometer (KYMERA-328I-B2, Andor technology LTD).

Transient photovoltage (TPV) and photocurrent (TPC) measurement. The lifetime of carriers can be measured by the transient photovoltage measurement. The background illumination was provided by a normal LED light source, and pulsed light was provided by arbitrary wave generator (AFG322C, Tektronix). The photovoltage traces were registered by the oscilloscope (AFG322C, Tektronix). The photocurrente traces were registered with the resistance of 50 Ω, switching open-circuit mode to short-circuit mode. The integrated TPC signal provides a measure of the total charge generated by the laser pulse (ΔQ). Empirically, the differential capacitance values are found to follow the exponential dependence on the open-circuit voltage given by $C = \frac{\Delta Q}{\Delta V} = C_0 exp(\gamma V_{OC}) + D$, and so the charge-carrier density

as a function of V_{OC} is given by treating the device as a parallel-plate capacitor and integrating with respect to voltage, as $n = \frac{1}{Aed}\int_{-\infty}^{V_{OC}} C_0 \exp(\gamma V) dV$, where A is the active layer area, and d is the active layer thickness. Then, the recombination rate coefficient can be determined, which is defined by $k(n) = \frac{1}{\tau(n)n}$.

Impedance Spectroscopy. Impedance measurements were carried out by illumination with a 1.5G illumination source (1000 W m-2) using a Solar Simulator. Impedance spectra were measured for different light intensities by applying a small voltage perturbation (10 mV rms) at frequencies from 8 MHz to 50 Hz, for different bias voltages. To measure in open circuit voltage conditions, a bias voltage equals to V_{OC} at each light intensity was applied. These measurements were performed with LCR-IM3536 equipped with a frequency analyzer module, always at room temperature. The data was collected for 10 average. Recombination resistance and chemical capacitance were directly extracted from the low-frequency region.

Supplementary Tables

Table S1. Photovoltaic parameters with additional AQx-1 for ternary devices under illumination of AM 1.5G, 100 mW/cm^2.[a]

PM6:Y6:AQx-1	V_{OC} (V)	J_{SC} (mA·cm^{-2})	FF (%)	PCE[b] (%)
1:1.2:0	0.841±0.002	25.47±0.16	76.39±0.81	16.36±0.12
1:1:0.2	0.853±0.003	26.45±0.15	77.86±0.54	17.61±0.09
1:0.6:0.6	0.861±0.002	25.71±0.21	76.52±1.12	16.88±0.22
1:0.2:1	0.869±0.004	24.13±0.19	74.02±0.77	15.44±0.19
1:0:1.2	0.875±0.001	22.68±0.28	72.22±0.93	14.33±0.28

[a]The device architecture is ITO/PEDOT:PSS/PM6:Y6:AQx-1/PFNDI-Br/Ag; D:A=1:1.2, D concentration = 6.5 mg/ml in CF with 0.5% 1-CN, following with 85°C TA treatment. [b]The average values are obtained from over 40 devices.

Table S2. Photovoltaic parameters with additional AQx-2 for ternary devices under illumination of AM 1.5G, 100 mW/cm^2.[a]

PM6:Y6:AQx-2	V_{OC} (V)	J_{SC} (mA·cm^{-2})	FF (%)	PCE[b] (%)
1:1.2:0	0.841±0.002	25.47±0.16	76.39±0.81	16.36±0.12
1:1:0.2	0.844±0.003	25.67±0.19	76.53±0.62	16.58±0.17

1:0.6:0.6	0.850±0.003	25.31±0.32	76.22±0.53	16.37±0.21
1:0.2:1	0.856±0.002	25.25±0.11	76.14±0.77	16..32±0.19
1:0:1.2	0.858±0.004	25.27±0.27	76.01±1.01	16.33±0.24

[a]The device architecture is ITO/PEDOT:PSS/PM6:Y6:AQx-2/PFNDI-Br/Ag; D:A=1:1.2, D concentration = 6.5 mg/ml in CF with 0.5% 1-CN, following with 85°C TA treatment. [b]The average values are obtained from over 40 devices.

Table S3. The mobility values derived from the SCLC results.

Devices	μ_e ($cm^2 \cdot s^{-1} \cdot V^{-1}$)	μ_h ($cm^2 \cdot s^{-1} \cdot V^{-1}$)
PM6:Y6	(6.685±0.360) ×10^{-4}	(8.646±0.219) ×10^{-4}
PM6:Y6:AQx-1	(9.924±0.260) ×10^{-4}	(12.172±0.392) ×10^{-4}
PM6:Y6:AQx-2	(8.842±0.372) ×10^{-4}	(9.753±0.221) ×10^{-4}
PM6:AQx-1	(6.459±0.418) ×10^{-4}	(7.483±0.272) ×10^{-4}

Table S4. Lifetime of the hole transfer process in blended films. The data was achieved through biexponential fitting.

**Blend**	**τ_1 (ps)**	**τ_2 (ps)**
PM6:Y6	0.4287±0.0165	17.352±0.889
PM6:Y6:AQx-1	0.3561±0.0141	12.914±0.439
PM6:Y6:AQx-2	0.3669±0.0166	13.734±0.605
PM6:AQx-1	0.5186±0.0256	26.348±1.84

Table S5. Urbach energy and DoS details for different blended devices.

Item	E_g^{PV} [eV]	E_U [meV]	N_t [*$10^{20}cm^{-3}$]	δ [meV]
PM6:Y6	1.352	26.25±0.43	3.30±0.32	56.5±1.0
PM6:Y6:AQx-1	1.355	25.18±0.36	3.95±0.48	54.4±0.8
PM6:Y6:AQx-2	1.353	26.22±0.24	3.42±0.39	56.2±0.7
PM6:AQx-1	1.357	25.74±0.32	2.93±0.46	54.1±0.9

Supplementary Figures

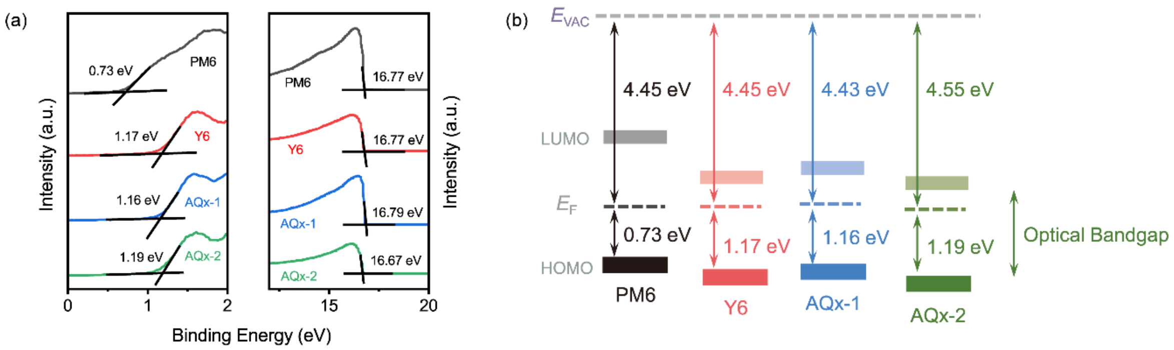

Figure S1. (a) UPS spectra corresponding to the secondary electron cutoff (right) and valence band maximum (left) of materials used in this study. The HOMO level referenced to the Fermi level (E_F) is extracted at the intensity onset of the frontier electronic structure region (left), while the work function is determined by the secondary electron cutoff (right) as calculated by (22.12-cutoff value) eV. (b) The scheme of materials' energy levels where the HOMO is determined by UPS measurements. The bandgap is extracted with the neat film absorption, and thus the LUMO level is determined.

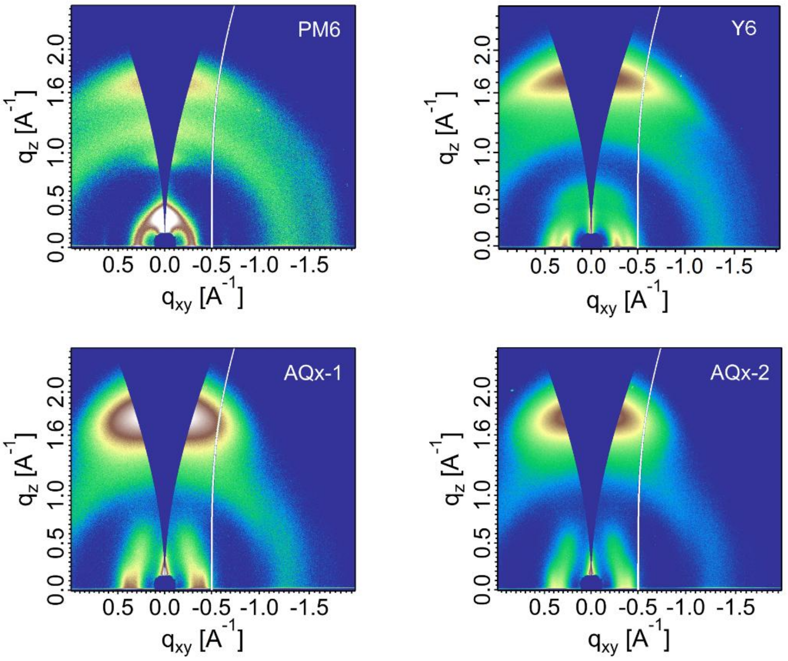

Figure S2. The 2D GIWAXS patterns of the neat films with CF as the processing solvent.

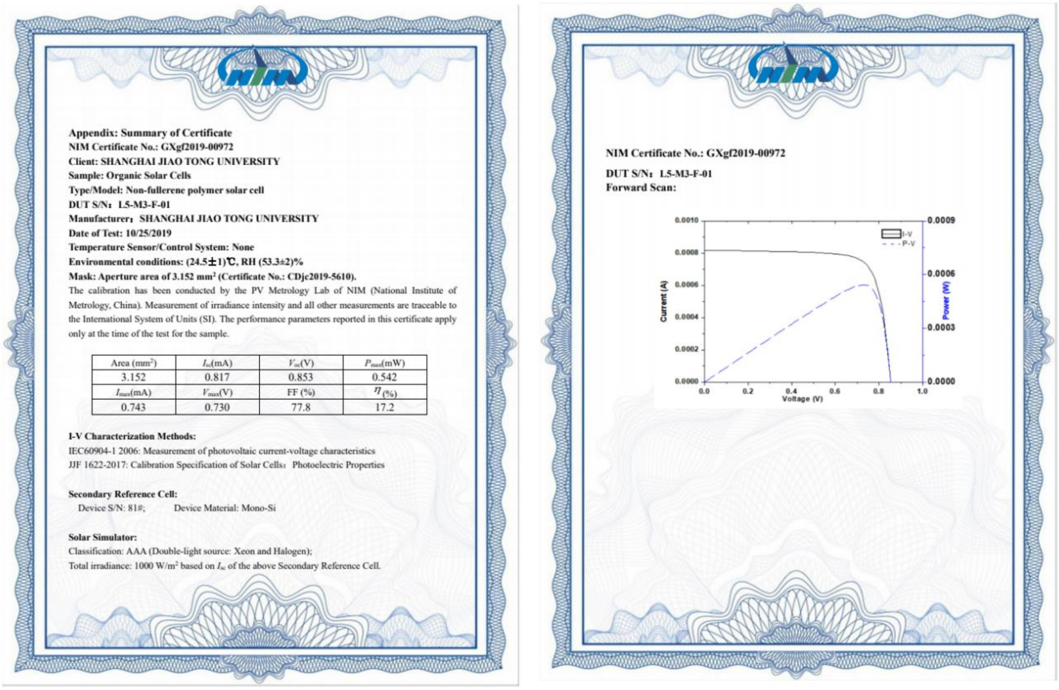

Appendix: Summary of Certificate
NIM Certificate No.: GXgf2019-00972
Client: SHANGHAI JIAO TONG UNIVERSITY
Sample: Organic Solar Cells
Type/Model: Non-fullerene polymer solar cell
DUT S/N： L5-M3-F-01
Manufacturer： SHANGHAI JIAO TONG UNIVERSITY
Date of Test: 10/25/2019
Temperature Sensor/Control System: None
Environmental conditions: (24.5±1)℃, RH (53.3±2)%
Mask: Aperture area of 3.152 mm^2 (Certificate No.: CDjc2019-5610).

The calibration has been conducted by the PV Metrology Lab of NIM (National Institute of Metrology, China). Measurement of irradiance intensity and all other measurements are traceable to the International System of Units (SI). The performance parameters reported in this certificate apply only at the time of the test for the sample.

Area (mm^2)	I_{sc}(mA)	V_{oc}(V)	P_{max}(mW)
3.152	0.817	0.853	0.542
I_{max}(mA)	V_{max}(V)	FF (%)	η (%)
0.743	0.730	77.8	17.2

I-V Characterization Methods:
IEC60904-1 2006: Measurement of photovoltaic current-voltage characteristics
JJF 1622-2017: Calibration Specification of Solar Cells： Photoelectric Properties

Secondary Reference Cell:
Device S/N: 81#; Device Material: Mono-Si

Solar Simulator:
Classification: AAA (Double-light source: Xeon and Halogen);
Total irradiance: 1000 W/m^2 based on I_{sc} of the above Secondary Reference Cell.

NIM Certificate No.: GXgf2019-00972
DUT S/N： L5-M3-F-01
Forward Scan:

Figure S3. Original images of the OSC certificate results by the National Institute of Metrology (NIM) in China. The PM6:Y6:AQx-1 device is measured with a mask of 0.03152cm^2, giving a PCE of 17.20%.

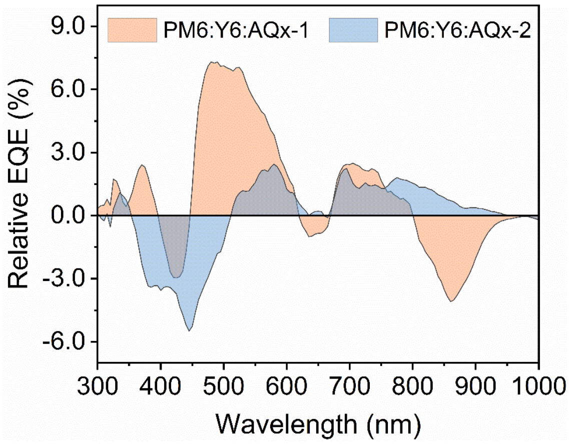

Figure S4. relative EQE value compared to that of PM6:Y6

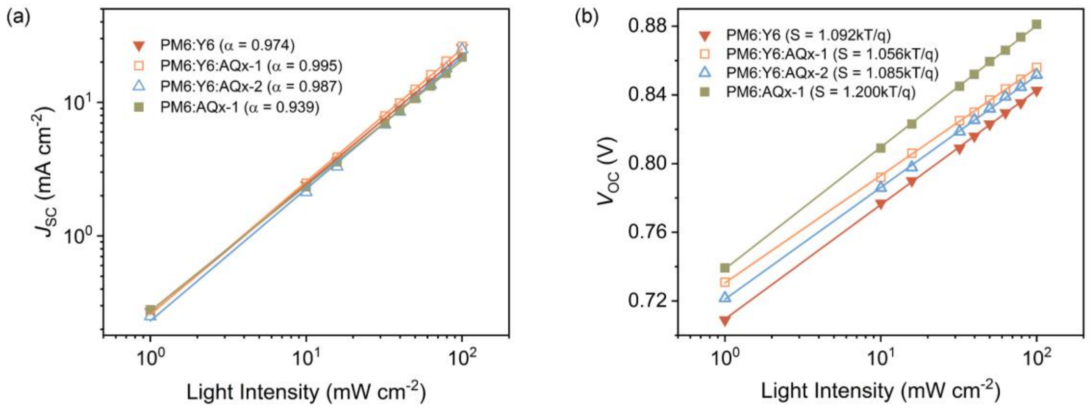

Figure S5. (a) Characteristics of J_{SC} versus light intensity, and (b) V_{OC} versus light intensity for binary and ternary devices.

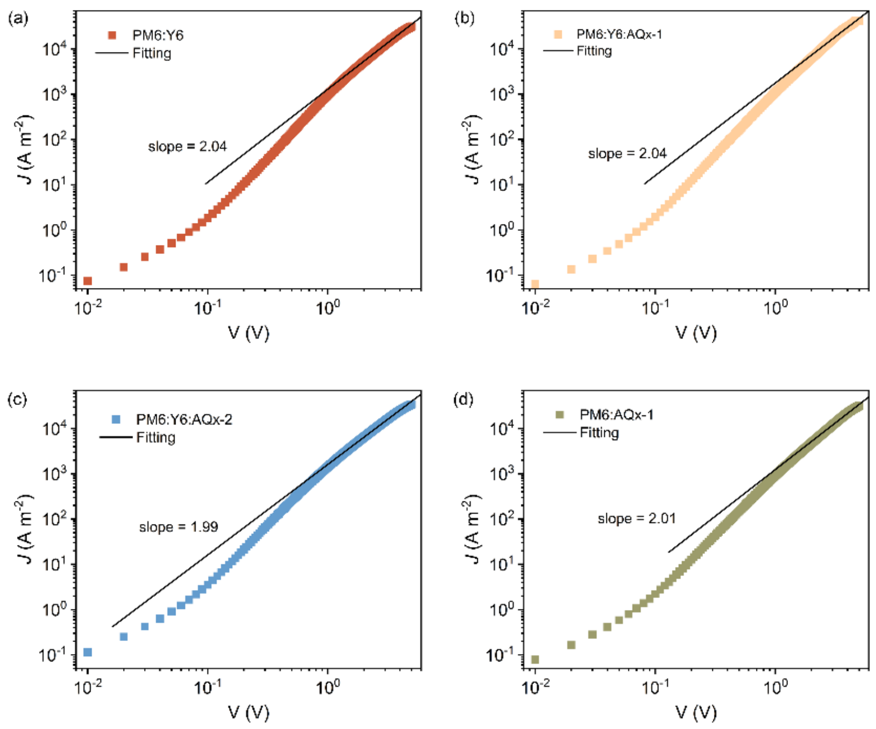

Figure S6. SCLC measurement electron-only devices. The black lines are the linear fitting, and the extracted intercept of the log-log plot could be used to calculate the mobility.

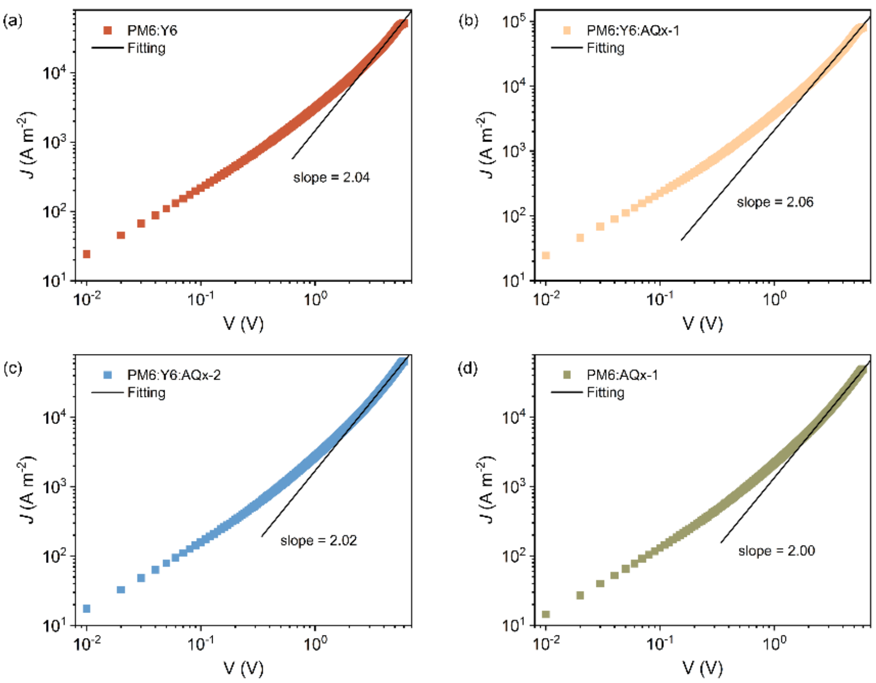

Figure S7. SCLC measurement hole-only devices. The black lines are the linear fitting, and the extracted intercept of the log-log plot could be used to calculate the mobility.

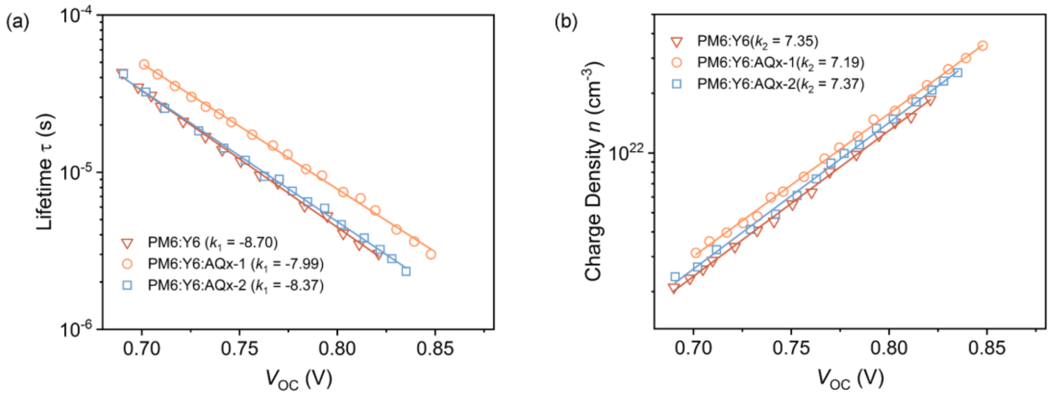

Figure S8. (a) Lifetime and (b) density of charge-carriers under different V_{OC} conditions.

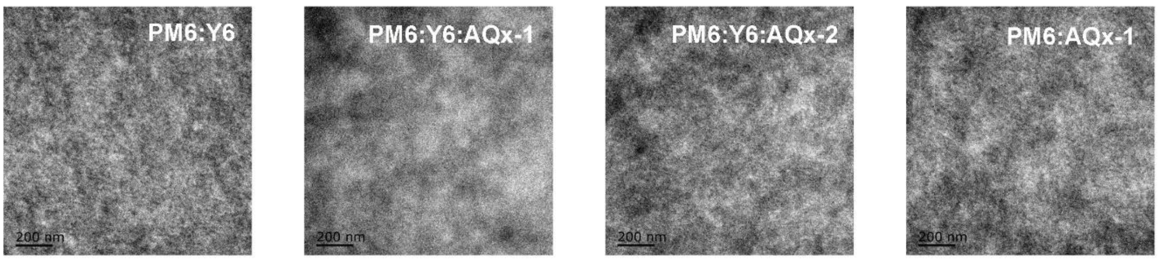

Figure S9. Transmission electron microscopy (TEM) of different blended films.

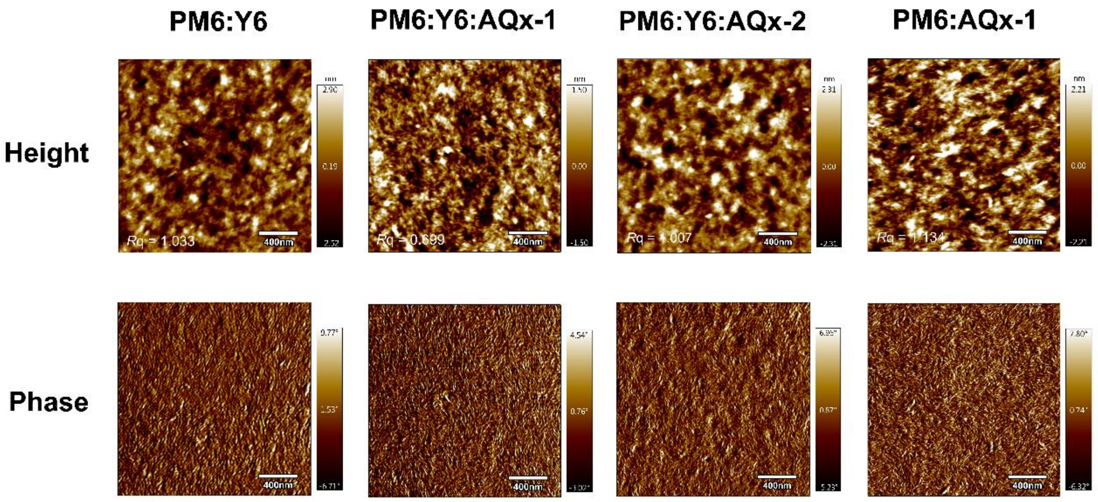

Figure S10. The AFM height and phase images of different blended films.

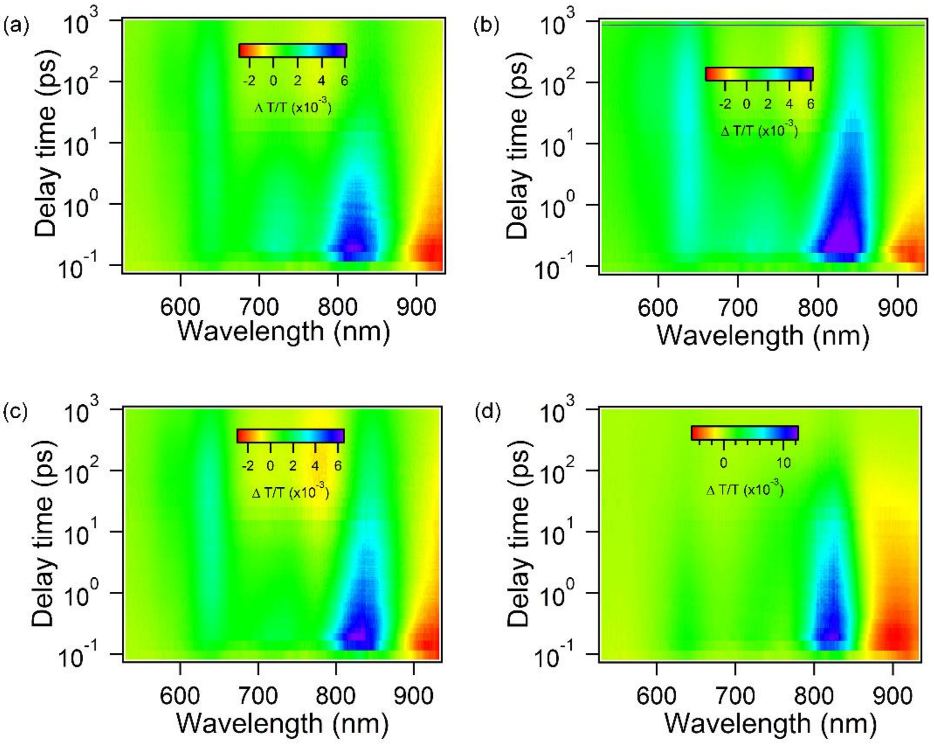

Figure S11. 2D Color plot of fs Transient absorption spectra of blended film under 750 nm excitation with a fluence below 10 μJ/cm^2 (a) PM6:Y6, (b) PM6:Y6:AQx-1, (c) PM6:Y6:AQx-2, and (d) PM6:AQx-1.

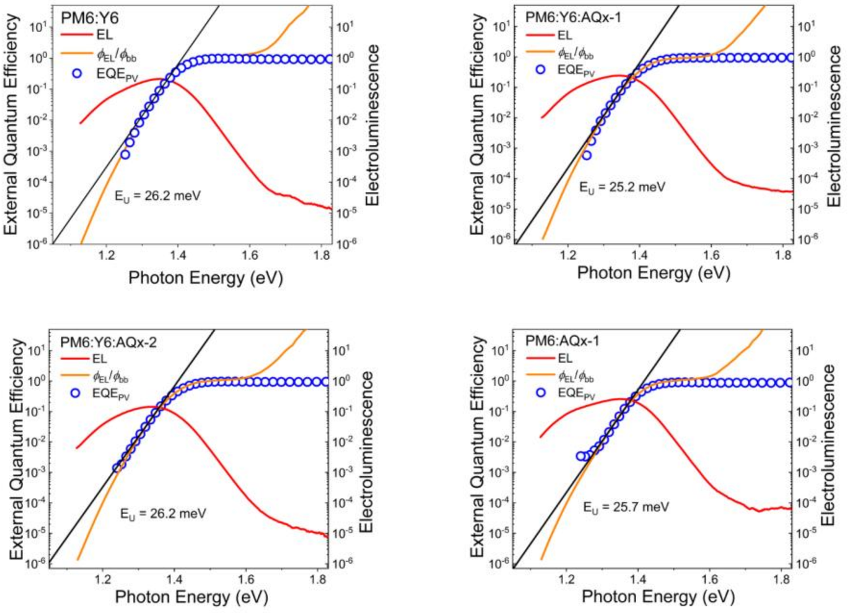

Figure S12. The semilogarithmic plots of the normalized EL (red solid line), normalized EQE (blue open circles) and the division of electroluminescence (Φ_{EL}) and black-body emission (Φ_{bb}) (orange solid line) of the binary and ternary devices. The black solid line is the urbach energy fitting.

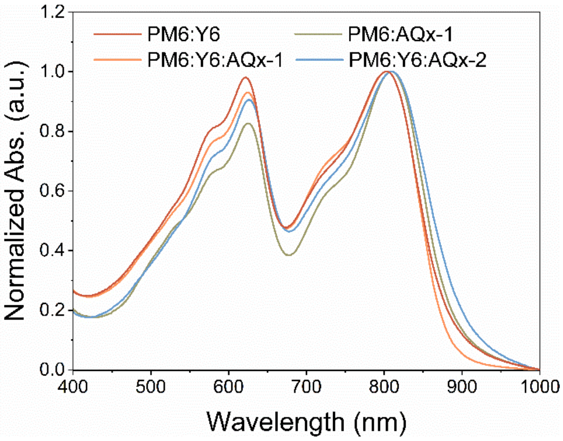

Figure S13. The normalized UV-vis absorption spectra of the BHJ blends. The absorption tail of the blends are 880.7nm, 879.4nm, 905.1nm and 892.1nm for PM6:Y6, PM6:Y6:AQx-1, PM6:Y6:AQx-2, and PM6:AQx-1, respectively.

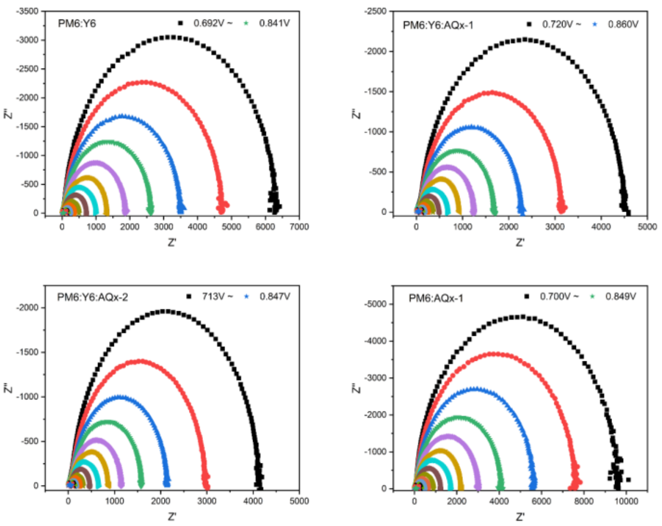

Figure S14. The Impedance spectra measured for different light intensities by applying a small voltage perturbation (10 mV rms) at frequencies from 8 MHz to 50 Hz, for different bias voltages.

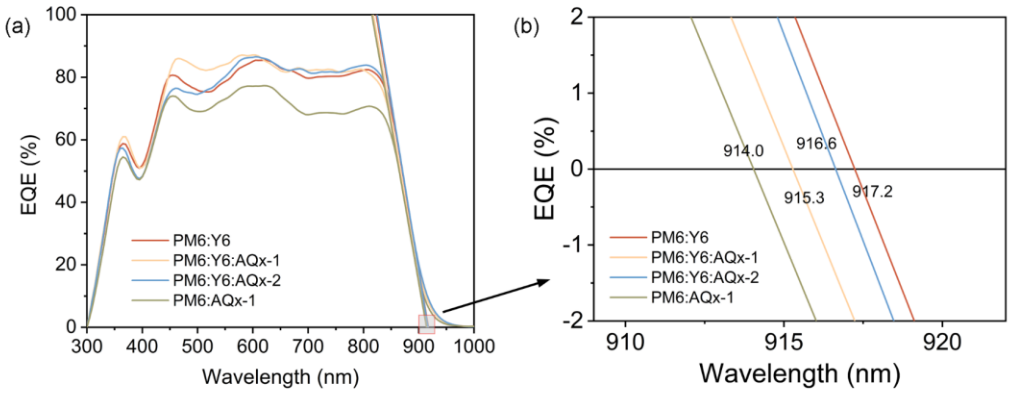

Figure S15. Fitting the onset of the EQE spectra to extract the E_g^{PV}.
